# Co-Administration of Propionate or Protocatechuic Acid Does Not Affect DHA-Specific Transcriptional Effects on Lipid Metabolism in Cultured Hepatic Cells

**DOI:** 10.3390/nu12102952

**Published:** 2020-09-26

**Authors:** Francesca Danesi, Bjørk D. Larsen, Mattia Di Nunzio, Ronni Nielsen, Dario de Biase, Veronica Valli, Susanne Mandrup, Alessandra Bordoni

**Affiliations:** 1 Department of Agricultural and Food Sciences (DISTAL), University of Bologna, 47521 Cesena, Italy; francesca.danesi@unibo.it (F.D.); mattia.dinunzio@unibo.it (M.D.N.); valli.veron@gmail.com (V.V.); 2 Interdepartmental Center for Agri-food Industrial Research (CIRI Agrifood), University of Bologna, 47521 Cesena, Italy; 3 Department of Biochemistry and Molecular Biology, University of Southern Denmark, Syddansk Universitet, 5230 Odense M, Denmark; bdlarsen@bmb.sdu.dk (B.D.L.); ronni.n@bmb.sdu.dk (R.N.); s.mandrup@bmb.sdu.dk (S.M.); 4 Department of Pharmacy and Biotechnology (FABIT), University of Bologna, 40138 Bologna, Italy; dario.debiase@unibo.it

**Keywords:** docosahexaenoic acid, DHA, propionic acid, 3,4-dihydroxybenzoic acid, bioactives, transcriptomics, RNA sequencing, HepG2, fatty acid synthesis, steroid synthesis

## Abstract

Long-chain n-3 polyunsaturated fatty acids (n-3 LC-PUFAs) are collectively recognized triglyceride-lowering agents, and their preventive action is likely mediated by changes in gene expression. However, as most studies employ fish oil, which contains a mixture of n-3 LC-PUFAs, the docosahexaenoic acid (DHA)-specific transcriptional effects on lipid metabolism are still unclear. The aim of the present study was to further elucidate the DHA-induced transcriptional effects on lipid metabolism in the liver, and to investigate the effects of co-administration with other bioactive compounds having effects on lipid metabolism. To this purpose, HepG2 cells were treated for 6 or 24 h with DHA, the short-chain fatty acid propionate (PRO), and protocatechuic acid (PCA), the main human metabolite of cyanidin-glucosides. Following supplementation, we mapped the global transcriptional changes. PRO and PCA alone had a very slight effect on the transcriptome; on the contrary, supplementation of DHA highly repressed the steroid and fatty acid biosynthesis pathways, this transcriptional modulation being not affected by co-supplementation. Our results confirm that DHA effect on lipid metabolism are mediated at least in part by modulation of the expression of specific genes. PRO and PCA could contribute to counteracting dyslipidemia through other mechanisms.

## 1. Introduction

Atherogenic dyslipidemia, i.e., low HDL cholesterol (HDL-c) and high triglycerides (TGs) blood level, is a diagnostic criterion for the metabolic syndrome (MetS). The primary causes of MetS are poor diet, obesity, and physical inactivity, and evidence indicates that a combination of lifestyle modifications with effective weight loss and drug therapy may serve as treatment. Lipid-lowering agents and LDL-lowering standard drugs, such as statins and ezetimibe, modify atherogenic dyslipidemia and reduce cardiovascular risk in patients with MetS [1] but the search for novel methods of preventing and treating dyslipidemia is still ongoing since current therapies induce various side effects [2]. Some nutritional agents have been therefore suggested as an alternative strategy. Several clinical trials have been carried out to find out the potential benefits of long-chain n-3 polyunsaturated fatty acids (n-3 LC-PUFAs) in metabolic disorders and cardiovascular prevention [3,4,5,6]. Eicosapentaenoic acid (EPA, C20:5 n-3) and docosahexaenoic acid (DHA, C22:6 n-3) are grouped together as n-3 LC-PUFAs but there is substantial evidence suggesting that the individual fatty acids may have distinct effects [7]. The EU Register contains three authorized Article 13.1 health claims for EPA and DHA together: the claims report maintenance of normal blood pressure, normal blood TG levels and the normal function of the heart. Three additional Article 13.1 claims reporting maintenance of normal brain function, normal vision and normal blood TG levels have been approved for DHA alone [8]. The effects of n-3 LC-PUFAs on lipid metabolism are likely mediated by changes in gene expression. Overall, n-3 LC-PUFAs bind to peroxisome proliferator-activated receptors (PPARs) [9] and consequently have the potential to lower plasma TG level and increase HDL-c level [10]. However, it is still unclear whether there are different molecular targets/pathways with EPA and DHA as most studies employ them as mixtures in purified forms as capsules or oily fish diet/supplements. Emerging evidence indicates that DHA is more efficacious than EPA at reducing blood TGs and does so in part via differential regulation of lipogenic and TG clearance pathways [11]. However, additional studies are needed to further clarify how EPA and DHA individually regulate hepatic pathways related to lipid metabolism. The first aim of the present study was to further elucidate the DHA-specific transcriptional effects on lipid metabolism.

Other nutritional agents have been reported to favorably modulate lipid metabolism. Dietary fibers, via their digestion or fermentation by intestinal microbiota may affect cholesterol metabolism by acting on bile acids and affecting lipid absorption [12]. However, the precise mechanisms of action of dietary fibers remain unclear and could be mediated by a transcriptional modulation [13] through metabolites produced by the gut microbiota such as short-chain fatty acids [14]. Short-chain fatty acids, mainly propionate (PRO), have been reported to affect cholesterol metabolism in the liver [15].

In a systematic review and meta-analysis of 32 clinical studies, it was revealed that anthocyanin-rich food possessed favorable effects on controlling the LDL level and can exert promising preventive and protective effects against cardiometabolic disorders. The major proposed mechanism of action for anthocyanins are antioxidant action, suppression of the nitric oxide production and induction of Nrf2 transcription triggering heme oxygenase-1 expression [16]. Notwithstanding, their direct modulation of lipid metabolism could have a role. Metabolites of cyanidin 3-glucoside, which is present in 90% of fruits, were shown to increase cholesterol efflux, ABCA1 expression, PPARα, LXRα in HK-2 cells [17] and to reduce lipid synthesis in the liver and white adipose tissue in mice [18].

As DHA, PRO and anthocyanins have positive effects on dyslipidemia through different molecular mechanisms of action, their possible additive or synergistic effect deserves attention. Therefore, once the DHA-specific effects were evidenced on the transcriptome, our second aim was to verify if the co-supplementation with PRO or with protocatechuic acid (3,4-dihydroxybenzoic acid, PCA), the main metabolite of cyanidin-3-glucoside, could amplify DHA effect. DHA, PCA and PRO were supplemented at concentrations within the physiological range in human blood, which were established as the highest non-toxic concentration in previous work [19]. Treatment lasted for 6 or 24 h, then we mapped the global transcriptional changes and correlated them with the modification observed in the cell lipid environment.

## 2. Materials and Methods

### 2.1. Materials

Dulbecco’s modified Eagle’s medium (DMEM) and Dulbecco’s phosphate-buffered saline (DPBS) were purchased from Lonza (Basel, Switzerland). All other chemicals, reagents, and solvents were purchased from Sigma-Aldrich Co. (St. Louis, MO, USA) unless otherwise stated.

### 2.2. HepG2 Cell Culture and Supplementation

HepG2 human hepatoma cells were maintained at 37 °C, 95% air, 5% CO_2_ in DMEM supplemented with 10% (*v*/*v*) fetal bovine serum (FBS), 100 U/mL penicillin and 100 µg/mL streptomycin [19]. Once a week, cells were split 1:20 into a new 75 cm^2^ flask, and the medium was refreshed. Cells were seeded in 12-well plates at 6 × 10^5^ cells/mL concentration, and after 24 h, they were incubated for 6 or 24 h with the bioactive compounds as previously described [20]. DHA was dissolved in 100% ethanol and complexed to bovine serum albumin (BSA). The DHA-BSA complex was prepared fresh each time at a final BSA concentration of 0.5% in serum-free DMEM. PCA was dissolved in dimethyl sulfoxide (DMSO) acidified with HCl to pH 2, while PRO was dissolved in water. The final concentration of ethanol and DMSO was kept below 0.1% in serum-free DMEM. Not supplemented, control cells (Ctrl) received corresponding amounts of BSA, ethanol and DMSO. DHA, PRO and PCA concentrations used in the study (50 μM DHA; 70 μM PRO; 20 μM PCA) were not cytotoxic to HepG2 cells as evidenced by different assays in preliminary experiments [19].

### 2.3. Lipid Extraction and Fatty Acid Composition Analysis

After 6 h or 24 h of supplementation medium was removed, cells were washed twice with warm DPBS, incubated with trypsin-EDTA for 2 min to remove adherent cells, and suspended in DMEM supplemented with 10% (*v*/*v*) FBS. Cell total lipids were extracted, and fatty acids were methylated as previously described [20]. Total fatty acid composition (as methyl esters) was determined by gas chromatography (GC) according to Ghini et al. [20]. The gas-chromatographic peaks were identified based on their retention time ratios relative to methyl stearate and predetermined using authentic samples. Gas chromatographic traces and quantitative evaluations were obtained using a Total-Chrom Navigator (version 6.2.1) (PerkinElmer, Shelton, CT, USA).

### 2.4. Isolation of RNA, RNA Sequencing (RNA-seq) and Quantitative Real-Time PCR (RT-qPCR)

After 6 or 24 h incubation, cells were gently washed twice with 1 mL of warm DPBS and scraped-off in 1 mL of ice-cold DPBS. Cell pellets were collected by centrifugation at 250× *g* for 5 min at 4 °C, and the supernatant was removed. Total RNA was isolated using the Direct-zol™ RNA MiniPrep (Zymo Research Corporation; Irvine, CA, USA) according to the manufacturer’s protocol. Three independent cell cultures were performed, with three replicates from each experiment. Replicates from the same experiment were pooled together and diluted to 40 ng/µL. The quantity and quality of RNA were assessed using the NanoDrop ND-2000 spectrophotometer (Thermo Fisher Scientific; Wilmington, DE, USA) and the Fragment Analyzer™ Automated CE system (Agilent; Santa Clara, CA, USA). Samples were frozen at −80 °C until further use.

The mRNA enrichment, library construction and amplification were performed according to Illumina TruSeq RNA Library Prep kit v2 using the manufacturer’s instructions and sequenced on the Illumina HiSeq1500 platform. Sequencing reads were aligned to the human genome (hg19/GRCh37) using STAR [21] and processed using iRNA-seq pipeline [22]. Pathway analyses were performed using the KEGG database [23,24,25]. Other analyses of genome-wide data were performed using R Project for Statistical Programming plus additional packages.

Reverse-transcription and RT-qPCR were performed as previously described [26,27]. For sequences of forward and reverse primers, see Table S1. All oligonucleotides used in this study were synthesized by Integrated DNA Technologies (Leuven, Belgium). cDNA was subjected to quantitative real-time PCR analysis using the CFX Connect™ Real-Time PCR Detection System (Bio-Rad Laboratories; Hercules, CA, USA), according to QuantiTect SYBR Green RT-PCR kit (Qiagen; Hamburg, Germany). Samples were run in duplicate using the following program: initial denaturation at 95 °C for 15 min, followed by 40 cycles of 94 °C for 15 s, 60 °C for 30 s, and 72 °C for 30 s.

### 2.5. Intracellular Triglyceride (TG) Content and Cholesterol Secretion

HepG2 cells were seeded as reported above and supplemented with the bioactives for 6 or 24 h in DMEM without red phenol. After 6 or 24 h, media were collected, centrifuged at 3000× *g* for 3 min, and supernatants stored at −80 °C until analysis. Cells were washed twice with ice-cold PBS and lysed in 500 µL of lysis buffer (0.25% Nonidet P-40 in DPBS). The homogenate was centrifuged at 14000× *g* for 15 min at 4 °C.

Intracellular TG content was measured in the cell pellet using the PicoProbe Triglyceride Quantification Assay Kit (Abcam; Cambridge, UK) according to the manufacturer’s protocol, using a M200 microplate reader (Tecan; Salzburg, Austria). Results were expressed as the percentage of the value obtained in control cells, assigned as 100%.

Total cholesterol was quantified in the media using the Cholesterol assay kit (Abcam; Cambridge, UK) according to the manufacturer’s instructions with the Infinite M200 microplate reader (Tecan). Results were expressed as µg of total cholesterol per mL of medium and normalized for mg of protein. Protein concentration was determined according to Bradford [28] using BSA as standard.

### 2.6. Statistical Analysis

#### 2.6.1. Biochemical and RT-qPCR Data Analysis

Resulting data on fatty acid composition, TG content, cholesterol concentration, and RT-qPCR data are given as means ± standard deviation (SD). Statistical significance among treatments was determined by the one-way analysis of variance (ANOVA) followed by Tukey’s HSD test, considering *p* < 0.05 as significant.

#### 2.6.2. RNA-Seq Analysis

The clustering was performed by sequential K-means and C-means clustering to obtain genes with high membership (Mfuzz package for R). Pathway analyses were performed using ClusterProfiler package for R based on the KEGG database [23,24,25]. Statistical significance among treatments was determined by the one-way ANOVA followed by Tukey’s HSD test, as appropriate. Differences with *p* < 0.05 were considered significant.

## 3. Results and Discussion

**Figure 1:**
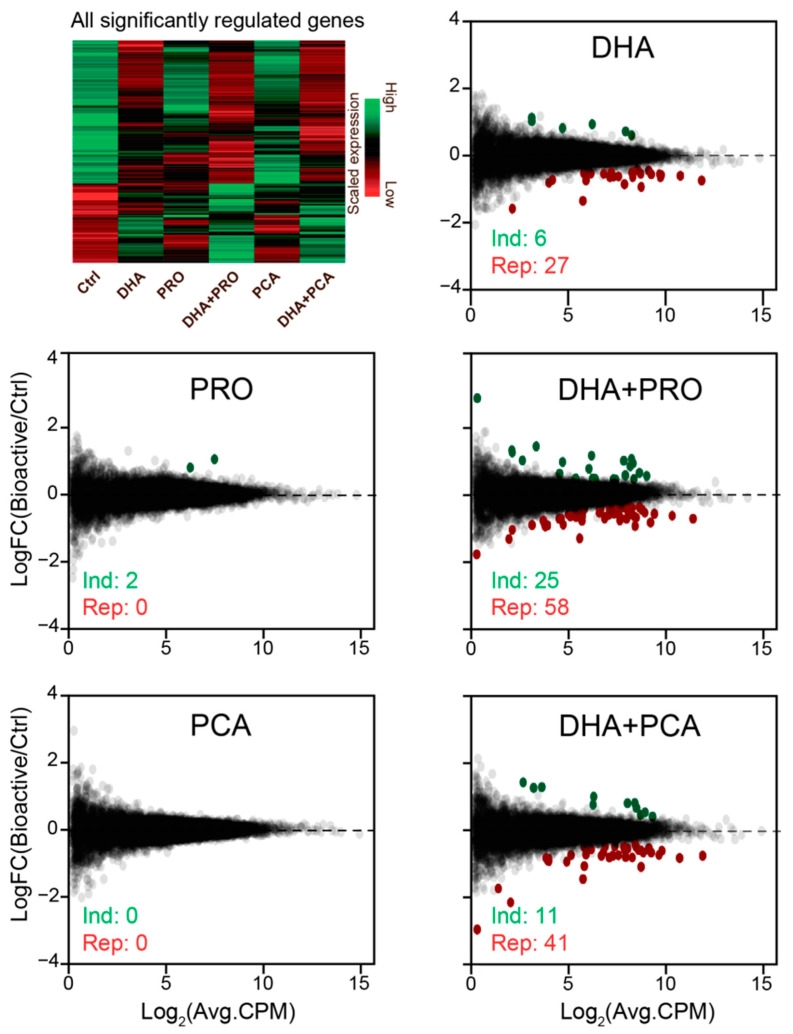
Global effects of docosahexaenoic acid (DHA), propionate (PRO), protocatechuic acid (PCA) or combinations hereof on gene expression in HepG2 cells. Heatmap representing the effects of the bioactives on the expression of genes found to be regulated by at least one of the bioactive conditions or combinations thereof. The expression is illustrated as scaled tag counts (using scale function in R) clustered by bioactive effects. Data in this figure represents 3 biological replicates. MA plots showing the transcriptional effect of 24 h supplementation with bioactives. LogCPM = log_2_ normalized sequence counts per million (CPM), LogFC = log_2_ fold change (FC) in expression between control and bioactive treatment. Green and red dots represent significantly induced and repressed genes (adjusted *p*-value ≤ 0.05, log_2_CPM > 0, log_2_FC > 0 (induced) or log_2_FC < 0 (repressed)), respectively.

To investigate the direct effect of the bioactives DHA, PRO and PCA on human liver cells, we treated HepG2 cells with the individual bioactives, and combination thereof or vehicle for 6 or 24 h, and determined the effect on the transcriptome by RNA-seq (Figure 1). DHA had a greater impact on transcriptional regulation than PRO and PCA, and the effect was shown to be time-dependent. This could reflect the time-dependent uptake of supplemented fatty acid, which was not influenced by co-administration with PRO and PCA (Table S2). The comparison of RNA-seq from HepG2 cells exposed to the bioactives for 6 and 24 h showed similar trends for activation and repression of gene clusters, but the fold regulation was generally greater at 24 h (Figure S1). Further data analysis was therefore focused on the 24 h timepoint. After 24 h supplementation, PRO and PCA alone had very little effect on the transcriptome. DHA repressed more genes than it activated, and the number of significantly regulated genes increased after co-administration of DHA and PRO or PCA (Figure 1).

**Figure 2:**
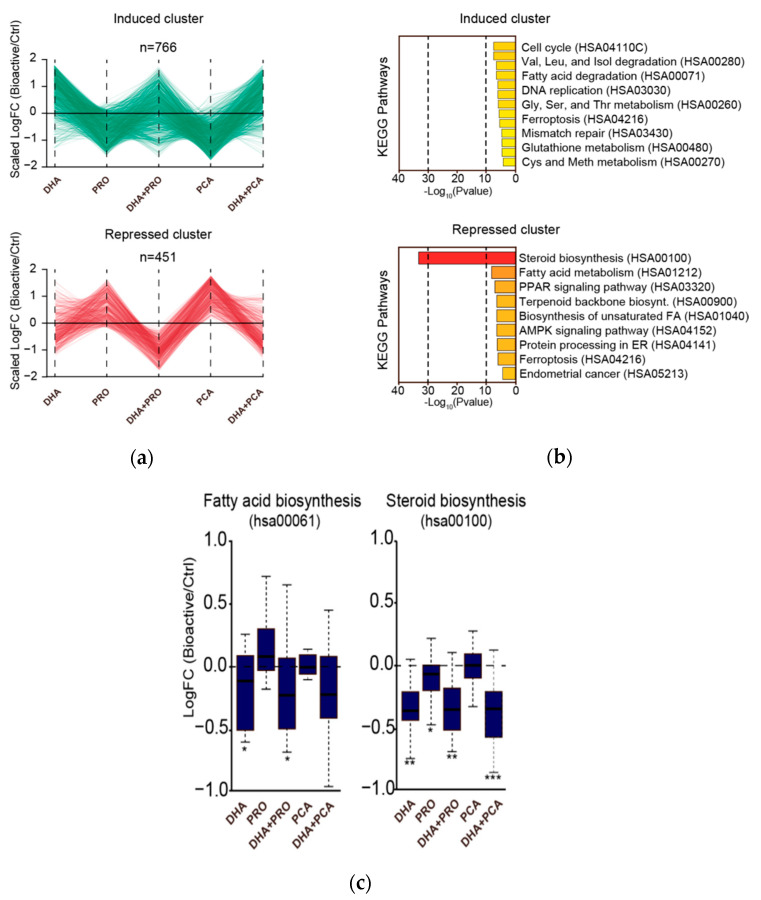
(**a**) Line plots representing scaled log_2_ fold changes (FC) following bioactive treatment of all expressed genes (LogCPM > 2) affected by DHA supplementation (abs(LogFC(DHA/Ctrl) ≥ 0)) and assigned to a cluster. The clusters were obtained by initial K-means clustering followed by C-means clustering (using Mfuzz package for R) to obtain genes with a high membership score (>0.8, fuzzification = 2); (**b**) Bar plots illustrating significantly (adjusted *p*-value ≤ 0.05) induced and repressed pathways within each cluster, respectively. Performed using enrichKEGG function in clusterProfiler package for R; (**c**) Boxplots illustrating the log_2_ fold change in expression of genes belonging to the KEGG pathways hsa00061 (fatty acid biosynthesis) and hsa000100 (steroid biosynthesis) following bioactive treatment (* *p* < 0.05, ** *p* < 0.005, *** *p* < 0.0005).

Clustering of the genes regulated by the bioactives revealed one cluster of DHA-repressed genes and a less well-defined cluster of DHA-induced genes (Figure 2a). Co-treatment with PRO increased the magnitude of DHA-induced regulation for both activated and repressed genes while co-treatment with PCA did not affect DHA-induced gene regulation.

Pathway analysis of the DHA-induced gene cluster (Figure 2b) showed enrichment of genes associated with cell cycle and DNA replication as well as pathways related to fatty acid and amino acid degradation. This could suggest DHA-promoted growth and energy turnover, in line with the previous study by Jump et al. [29]. However, given the rather low cluster membership of the DHA-induced genes and the lack of effects of DHA on cell numbers, viability and vitality, we argue that DHA did not regulate rate-limiting steps for cell cycle in HepG2 cells. Among significantly induced pathways, we found valine and threonine degradation and glutathione metabolism. This is consistent with metabolomics data obtained in parallel experiments showing significantly lower levels of the two amino acids and higher level of glutathione in HepG2 cells treated with DHA [20]. These results represent interesting, very specific and palpable links between DHA-induced transcriptional regulation and metabolic effects.

Pathway analysis of DHA-repressed genes showed an almost exclusive enrichment of the steroid biosynthesis pathway. Analysis of the genes in the steroid biosynthesis and the fatty acid biosynthesis pathways showed that both pathways were significantly repressed by DHA supplementation although the steroid biosynthesis pathway was most dramatically repressed (Figure 2c). The sterol biosynthesis pathway was also slightly repressed by PRO alone, while PCA showed no effect (Figure 2c). This clearly indicates that DHA interferes with anabolic lipid metabolism at a transcriptional level and that this primarily targets the cholesterol synthesis pathway.

Consistent with this, we found 3-hydroxy-3-methylglutaryl-CoA reductase gene (*HMGCR*) to be one of the transcripts most repressed by DHA. *HMGCR* encodes the rate-limiting enzyme of the cholesterol biosynthesis, and the repression of this gene by DHA in HepG2 is in agreement with results obtained in mouse and rat liver [30,31].

**Figure 3:**
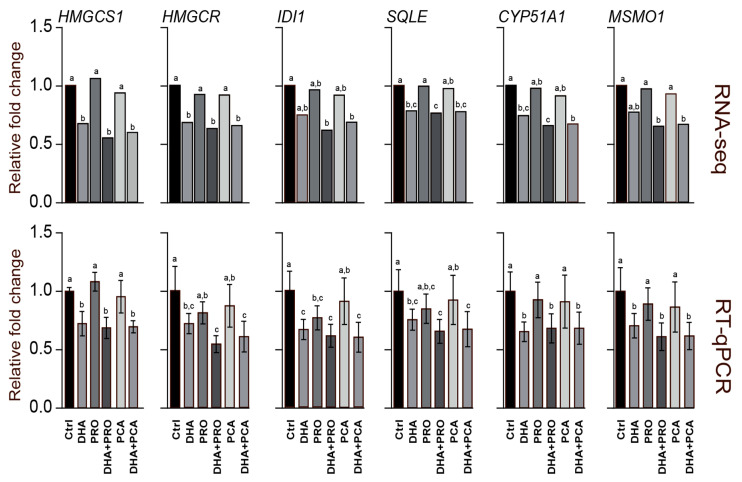
Comparison of transcriptional effects of 24 h exposure to DHA, PRO and PCA on genes involved in steroid biosynthesis (3-hydroxy-3-methylglutaryl-CoA synthase 1, *HMGCS1*; 3-hydroxy-3-methylglutaryl-CoA reductase, *HMGCR*; isopentenyl-diphosphate Δ isomerase 1, *IDI1*; squalene epoxidase, *SQLE*; cytochrome P450 family 51 subfamily A polypeptide 1, *CYP51A1*; methylsterol monooxygenase 1, *MSMO1*) mapped by RNA-seq and RT-qPCR. Data are represented as the mean fold change of relative expression compared to control cells. Statistical analysis was carried out using one-way ANOVA followed by Tukey’s HSD test. Different letters indicate statistical significance (*p* < 0.05).

To substantiate our findings, we validated the RNA-seq by RT-qPCR on a subset of genes known to be pivotal for the cholesterol biosynthesis pathway [32,33,34]. Beside the very good agreement between the data obtained by RNA-seq and RT-qPCR both at 6 (Figure S2) and 24 h (Figure 3) exposure to bioactives, these data further emphasize that PRO and PCA alone have little or no effect on the expression of genes associated with cholesterol biosynthesis, and that their co-administration with DHA do not further modulate the effect of DHA.

Consistent with the finding that DHA represses the expression of genes involved in fatty acid biosynthesis, the expression of the genes encoding acetyl-CoA carboxylase 1 (*ACACA*), the rate-limiting enzyme for fatty acid biosynthesis, and fatty acid synthase (*FASN*) was repressed by DHA while PRO and PCA had no effect. Similarly, genes encoding stearoyl-CoA desaturase 1 (*SCD1*), acyl-coenzyme A synthetase short-chain family member 2 (*ACSS2*) and ATP citrate lyase (*ACLY*) (Figure S3) were down-regulated by DHA only. This is in agreement with a previous report showing that the DHA supplementation decreases the expression of genes involved in fatty acid synthesis in liver cells [31]. Co-supplementation with PCA or PRO had no additional effect (Figure S3).

**Figure 4:**
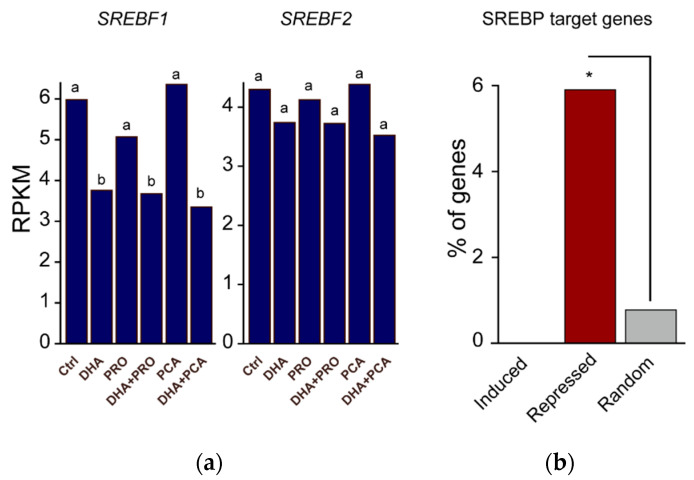
*SREBPF1*, *SREBPF2*, and SREBP-target gene expression. (**a**) *SREBF1* and *SREBF2* expression (RPKM: reads per kilobase per million mapped reads). Statistical analysis was carried out using one-way ANOVA followed by Tukey’s HSD test. Different letters indicate statistical significance (*p* < 0.05); (**b**) The relative percent enrichment of SREBP-target genes within each cluster and random genes (* *p* < 4 × 10^−9^, proportion test).

In liver, the suppression of SREBP-1 accounts for much of the decrease of de novo lipogenesis by dietary PUFAs, which are well-established regulators of SREBP-1, but not SREBP-2, nuclear abundance [29]. Among LC-PUFAs, DHA is indicated as the major suppressor of nSREBP-1 abundance in vivo, and the major component of this control is supposed to be at the post-translational level [35]. Notably, we observed a marked downregulation of *SREBF1* mRNA by DHA (Figure 4a), indicating a strong transcriptional component in the repression of SREBP1 activity. In contrast, we did not observe a pronounced downregulation of the mRNA levels of *SREBF2*, the master regulator of cholesterol synthesis, although genes involved in the synthesis were significantly repressed. It is worth noting that in cultured cells SREBP-2 and SREBP-1a are the most dominant isoforms [36]. SREBP-1a, which has a longer transcription-activating domain than the SREBP-1c isoform, is able to modulate expression of genes involved in both fatty acid and cholesterol biosynthesis [37]. Consistent with this, SREBP-target genes were enriched in the DHA-repressed cluster (Figure 4b).

**Figure 5:**
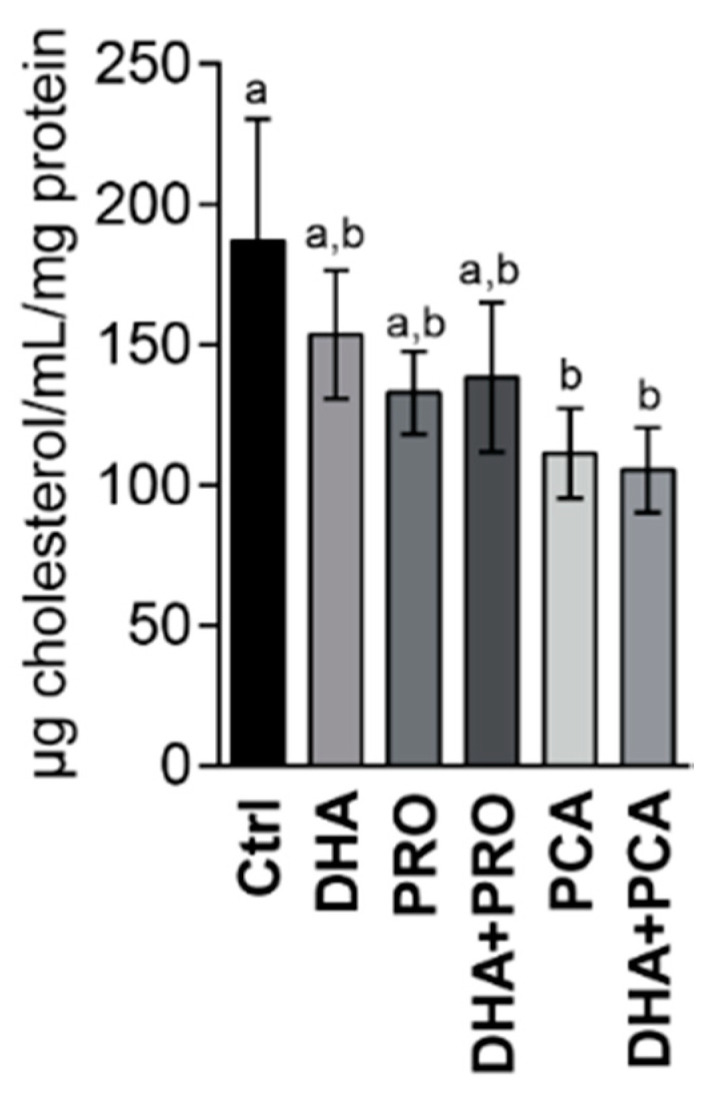
Cholesterol concentration in the media of control and supplemented cells after 24 h supplementation. Data are expressed as µg cholesterol/mL medium/mg protein in the well. Statistical analysis was carried out by the one-way ANOVA followed by Tukey’s HSD test. Different letters indicate statistical significance (*p* < 0.05).

Based on transcriptomic data, a reduced intracellular cholesterol concentration was expected in all DHA-supplemented cells. In contrast, parallel experiments evidenced a decreased cholesterol intracellular concentration only in cells supplemented with DHA alone [20]. In mammalian cells, cholesterol homoeostasis is stringently controlled, its level being maintained by a balance between uptake, efflux and endogenous synthesis. In hepatic cells, efflux deserves attention since hepatic biosynthesis largely contributes to cholesterol homeostasis in the body [38]. Supplementation with DHA alone did not modify cholesterol efflux, confirming that the reduced cholesterol concentration in DHA-supplemented cells was related to a decreased sterol biosynthesis. In PRO, and mainly PCA and DHA + PCA-supplemented cells, a decreased cholesterol efflux was observed (Figure 5), and let us hypothesize that the reduced cholesterol biosynthesis was counterbalanced by the reduced efflux, so keeping the concentration of cholesterol constant.

The absence of significant changes of the total fatty acid concentration in cells exposed to DHA compared to controls (Table S3) could be ascribable to the increased DHA uptake and incorporation into cell lipids (Table S2). Indeed, cells supplemented with DHA and DHA + PRO showed a trend to reduction in the concentration of total fatty acids other than DHA, and the concentration of total PUFAs other than DHA was significantly lower in cells supplemented with the fatty acid (Table S3). We speculate that DHA supplementation reduced fatty acid and PUFA synthesis, in agreement with transcriptomic data. Interestingly, the increased uptake of supplemented DHA was coupled to a transient increase in the intracellular level of TGs (Figure S4). Similar results were already reported in cultured LO2 hepatocytes [39] and cardiomyocytes [40] and could represent a protective mechanism in the early stage of DHA supplementation to avoid excess LC-PUFAs resulting in the generation of lipid peroxides and potential cytotoxicity [41].

## 4. Conclusions

In vitro studies, animal experiments, observational studies, and randomized clinical trials have examined the health effects of n-3 LC-PUFAs, which are known to affect a wide range of physiological functions in multiple tissues [42,43]. With unique chemical structures and three-dimensional configurations, n-3 LC-PUFAs influence multiple relevant molecular pathways, which individually or in sum might contribute to the observed effects on physiological risk factors and clinical events. A lingering issue in this field is that most studies of n-3 LC-PUFAs involve fish oil or mixtures of EPA and DHA without considering that each fatty acid could have distinct biological effects and tissue specific effects [44]. The present study was conducted to further characterize the independent, specific effect of DHA on the transcriptome of liver cells. Our aim was not to compare the effect of EPA and DHA supplementation, which is speculative since EPA is substantially converted to DHA within the human body [45], but to evidence the DHA-specific transcriptional targets.

Our results emphasize that DHA effects are mediated, at least in part, by modulation of gene expression and clearly indicate that it mainly interferes with anabolic lipid metabolism primarily targeting the cholesterol synthesis pathway. The observed impact of DHA treatment on the transcriptome is in line with recently published results in breast cancer cells, where 100 µM DHA strongly downregulated genes involved in the cholesterol biosynthesis pathway, especially after 24 h of treatment [46]. Of note, a strong transcriptional component in the repression of SREBP1 activity was observed, suggesting that the control of nSREBP-1 abundance by DHA is not solely at the post-translational level.

PRO and PCA alone had only a minor effect on the transcriptome, and although co-treatment with PRO increased the magnitude of DHA-induced regulation for both activated and repressed genes, it did not further modulate the effect of DHA on sterol and fatty acid biosynthesis in cultured hepatoma cells. This indicates that there is no major crosstalk between DHA and PRO and PCA at the level of transcriptional regulation. However, intervention studies are needed to exclude such as cross-talk in vivo, as PRO and PCA through pathways that are not represented by our in vitro model system. The herein reported study was conducted within the PATHWAY-27 EU project, in which the main objective was the formulation of food enriched with DHA, alone or in combination with oat β-glucan (source of PRO) and anthocyanins (source of PCA), and the validation of their effectiveness in the counteraction of MetS. PATHWAY-27 also included a pilot intervention study [47,48] and a larger intervention study (unpublished data). In the pilot study, DHA administration induced a metabolome perturbation that was influenced by the co-presence of the other bioactives [41], and both intervention trials evidenced that food enriched with DHA plus oat β-glucan or DHA plus anthocyanins offer a promising food-based strategy for MetS prevention.

### Availability of Supporting Data

The RNA sequencing data have been deposited in Gene Expression Omnibus with accession number GSE147718, which are available at link https://www.ncbi.nlm.nih.gov/geo/query/acc.cgi?acc=GSE147718. In the file name of the deposited dataset, propionate can be either PRO or PA (propionic acid).

## Supplementary Materials

The following are available online at https://www.mdpi.com/2072-6643/12/10/2952/s1, Figure S1: Gene expression change after 6 h and 24 h supplementation, Figure S2: Comparison of transcriptional effects of 6 h exposure to DHA, PRO and PCA on genes involved in steroid biosynthesis mapped by RNA-seq and RT-qPCR, Figure S3: Comparison of transcriptional effects on genes involved in fatty acid biosynthesis of bioactives on HepG2 cells mapped by RNA-seq, Figure S4: Intracellular triglyceride content in control and supplemented cells after 6 h and 24 h supplementation, Table S1: Primers used for RT-qPCR, Table S2: Fatty acid (FA) composition of HepG2 cells after 6 h and 24 h supplementation, Table S3: Total FA, PUFA, and DHA content in control and supplemented cells after 24 h supplementation.
