# Supplementary material for: Co-Administration of Propionate or Protocatechuic Acid Does Not Affect DHA-Specific Transcriptional Effects on Lipid Metabolism in Cultured Hepatic Cells
Source: Nutrients. 2020 Sep 26;12(10):2952. doi: 10.3390/nu12102952 (PMC7599819; doi:10.3390/nu12102952)
Supplement: Supplementary file 1 [file nutrients-12-02952-s001.pdf]

SUPPLEMENTARY DATA

SUPPLEMENTARY FIGURES

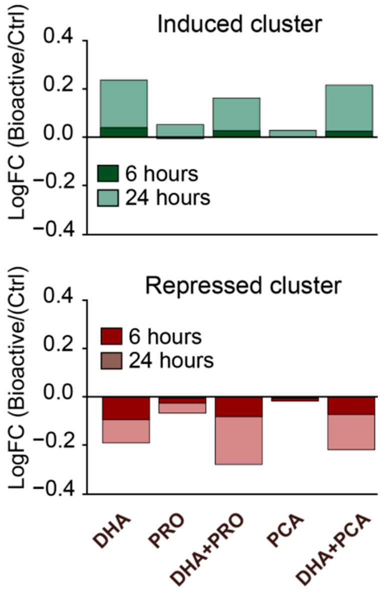

Figure S1. Gene expression change after 6 h and 24 h supplementation. Aggregate bar plots illustrating log_2 fold change (FC) in the expression of genes assigned to the induced (green) and repressed (red) clusters with 6 (saturated color) and 24 hours (pale color) bioactive supplementation.

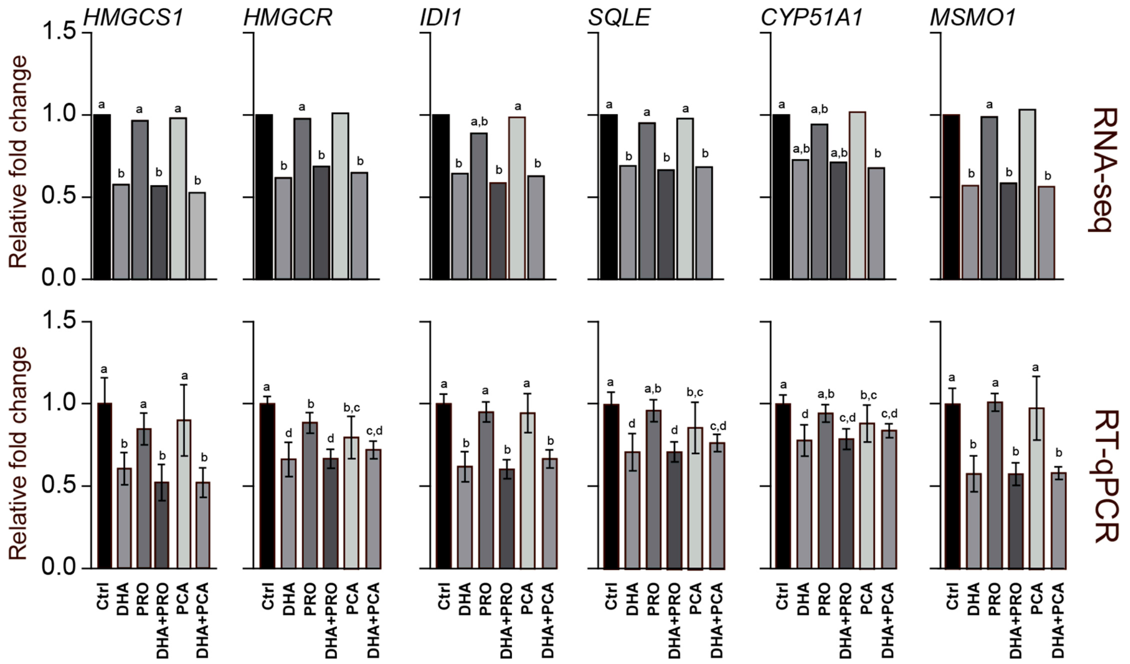

Figure S2. Comparison of transcriptional effects of 6 h exposure to DHA, PRO and PCA on genes involved in steroid biosynthesis (3-hydroxy-3-methylglutaryl-CoA synthase 1, *HMGCS1*; 3-hydroxy-3-methylglutaryl-CoA reductase, *HMGCR*; isopentenyl-diphosphate Δ isomerase 1, *IDI1*; squalene epoxidase, *SQLE*; cytochrome P450 family 51 subfamily A polypeptide 1, *CYP51A1*; methylsterol monooxygenase 1, *MSMO1*) mapped by RNA-seq and RT-qPCR. Data are represented as the mean fold change of relative expression compared to Ctrl cells. Statistical analysis was carried out using one-way ANOVA followed by Tukey's HSD test. Different letters indicate statistical significance ($p<0.05$).

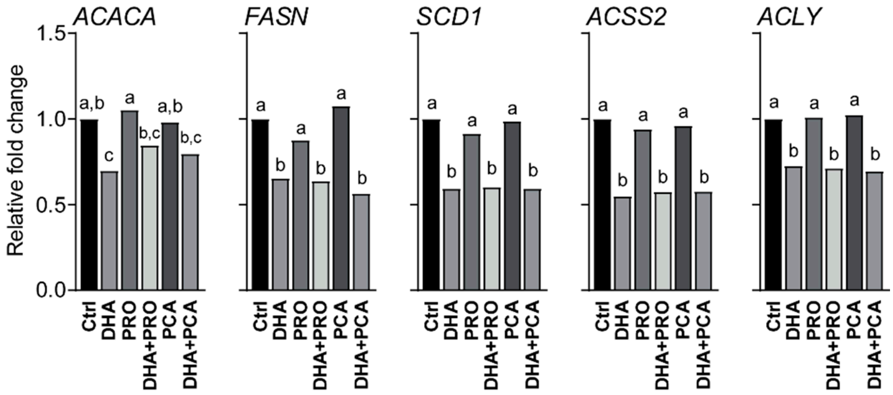

Figure S3. Comparison of transcriptional effects on genes involved in fatty acid biosynthesis (acetyl-CoA carboxylase 1, *ACACA*; fatty acid synthase, *FASN*; stearoyl-CoA desaturase 1, *SCD1*; acyl-coenzyme A synthetase short-chain family member 2, *ACSS2*; ATP citrate lyase, *ACLY*) of bioactives on HepG2 cells mapped by RNA-seq. Bar plots representing gene expression in Ctrl and supplemented cells after 24 h of supplementation. Data are represented as the mean fold change of relative expression compared to Ctrl cells. Statistical analysis was carried out using one-way ANOVA followed by Tukey's HSD test. Different letters indicate statistical significance ($p<0.05$).

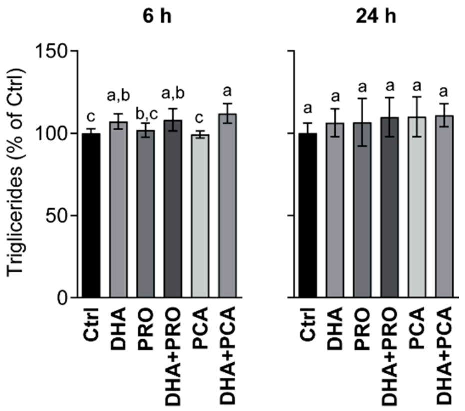

Figure S4. Intracellular triglyceride content in control (Ctrl) and supplemented cells after 6 h and 24 h supplementation. Data are expressed as the percentage of the value obtained in control cells (assigned as 100%). Statistical analysis was carried out using one-way ANOVA followed by Tukey's HSD test. Different letters indicate statistical significance ($p<0.05$).

SUPPLEMENTARY TABLES

Table S1. Primers used for RT-qPCR.

Gene name	GenBank accession number	Primer sequence
	**Reference genes**	
ACTB β-actin	NG_007992.1	F: ATGTGGCCGAGGACTTTGATT R: AGTGGGGTGGCTTTTAGGATG
GAPDH glyceraldehyde-3-phosphate dehydrogenase	NG_007073.2	F: AAGGTGAAGGTCGGAGTCAA R: AATGAAGGGGTCATTGATGG
HMBS hydroxymethylbilane synthase	NG_008093.1	F: ACCAAGGAGCTTGAACATGC R: GAAAGACAACAGCATCATGAG
SDHA subunit A of succinate dehydrogenase complex	NG_012339.1	F: TGGGAACAAGAGGGCATCTG R: CCACCACTGCATCAAATTCATG
	**Target genes**	
CYP51A1 cytochrome P450 family 51 subfamily A member 1	NG_007968.1	F: CCTTTGCCTAGTTTCAGACGC R: GACTGTCTGCGTTTCTGGATTG
HMGCR 3-hydroxy-3-methylglutaryl-CoA reductase	NG_011449.1	F: TGTCAGGGGTACGTCAGCTT R: AGGACACACAAGCTGGGAAG
HMGCS1 3-hydroxy-3-methylglutaryl-CoA synthase 1	NC_000005.10	F: CCAGTGGCAGAAAGAGGGAA R: GTCATTCAGCAACATCCGAGC
IDI1 isopentenyl-diphosphate Δ isomerase 1	NC_000010.11	F: ACTAACCACCTCGACAAGCAA R: TCCTTTCTCAATGTTCTCGTTCA
MSMO1 methylsterol monooxygenase 1	NG_042288.1	F: TCCAGCTGCCTTTGATTTGTG R: CAATGACTGCACAACCAAAGC
SQLE squalene epoxidase	NC_000008.11	F: AGGCGCAGAAAAGGAACCAA R: GCCAGCTCCCACGATGATAA

Table S2. Fatty acid (FA) composition of HepG2 cells (**a**) after 6 h and (**b**) after 24 h supplementation.

(**a**)

FA	Ctrl	50 µM DHA	70 µM PRO	50 µM DHA +70 µM PRO	20 µM PCA	50 µM DHA +20 µM PCA
3:0	0.10±0.13[a]	0.10±0.11[a]	0.19±0.14[a]	0.03±0.07[a]	0.05±0.05[a]	0.04±0.05[a]
14:0	1.94±0.58[a]	2.33±0.72[a]	1.53±0.77[a]	1.96±0.26[a]	1.70±0.77[a]	2.14±0.17[a]
16:0	33.54±2.71[a]	31.99±0.84[a]	32.29±0.59[a]	31.51±0.93[a]	32.32±1.13[a]	31.33±0.16[a]
16:1n-7	2.55±0.57[a]	2.70±0.56[a]	2.71±0.75[a]	2.59±0.27[a]	2.97±0.43[a]	2.93±0.17[a]
18:0	29.45±4.32[a]	25.66±2.54[a]	28.23±2.16[a]	25.93±1.42[a]	27.19±1.17[a]	25.38±2.39[a]
18:1n-9	9.49±3.33[a]	10.13±1.76[a]	10.72±1.72[a]	8.74±0.90[a]	10.27±1.67[a]	9.03±1.34[a]
18:1n-7	10.55±1.11[a,b,c]	8.32±0.83[c]	11.18±0.96[a,b]	8.91±0.97[b,c]	11.52±1.17[a]	9.07±1.12[b,c]
18:2n-6	0.40±0.13[a]	0.43±0.22[a]	0.42±0.17[a]	0.39±0.05[a]	0.41±0.07[a]	0.37±0.07[a]
18:3n-3	0.55±0.12[a]	0.67±0.15[a]	0.52±0.26[a]	0.51±0.04[a]	0.48±0.21[a]	0.49±0.05[a]
20:4n-6	9.72±2.43[a]	9.02±1.43[a]	10.97±0.99[a]	10.66±1.41[a]	11.65±1.54[a]	10.48±1.36[a]
20:5n-3	0.38±0.03[a]	0.40±0.08[a]	0.37±0.05[a]	0.66±0.72[a]	0.42±0.27[a]	0.32±0.03[a]
22:6n-3	1.34±0.45[b]	8.25±1.17[a]	0.86±0.39[b]	8.11±1.39[a]	1.02±0.28[b]	8.41±0.03[a]

(**b**)

FA	Ctrl	50 µM DHA	70 µM PRO	50 µM DHA +70 µM PRO	20 µM PCA	50 µM DHA +20 µM PCA
3:0	0.68±0.30[a]	1.12±1.15[a]	0.66±0.19[a]	1.18±1.48[a]	1.17±1.24[a]	0.71±0.48[a]
14:0	3.11±0.15[a]	3.37±0.82[a]	3.54±0.98[a]	3.85±0.36[a]	3.76±0.66[a]	3.68±0.40[a]
16:0	29.85±1.18[a]	28.45±2.13[a]	29.06±1.89[a]	28.09±1.02[a]	29.62±0.99[a]	28.34±0.52[a]
16:1n-7	4.62±0.83[a,b,c]	3.89±0.43[c]	5.70±1.050[a]	4.24±0.21[a,b,c]	5.62±1.01[a,b]	4.05±0.32[b,c]
18:0	27.10±5.36[a]	21.60±3.97[a,b]	18.15±3.63[b]	17.65±2.40[b]	17.48±1.72[b]	17.40±3.49[b]
18:1n-9	17.16±2.47[b]	14.48±1.25[b]	21.41±0.80[a]	15.53±0.80[b]	21.08±0.83[a]	15.45±1.04[b]
18:1n-7	10.88±1.63[b]	7.78±0.94[c]	13.63±0.24[a]	8.42±0.47[c]	13.68±0.43[a]	8.20±0.60[c]
18:2n-6	0.82±0.18[a]	0.41±0.05[b]	0.96±0.08[a]	0.44±0.02[b]	0.94±0.13[a]	0.41±0.02[b]
18:3n-3	0.86±0.16[a,b]	0.66±0.06[b]	1.05±0.09[a]	0.68±0.05[b]	1.06±0.11[a]	0.70±0.06[b]
20:4n-6	1.60±0.26[a]	1.39±0.39[a]	2.15±0.79[a]	1.87±0.36[a]	2.12±0.11[a]	1.98±0.46[a]
20:5n-3	0.69±0.60[a]	0.38±0.44[a]	0.71±0.84[a]	0.25±0.42[a]	0.64±0.82[a]	0.63±0.48[a]
22:6n-3	2.62±0.81[b]	16.48±3.01[a]	2.99±0.96[b]	17.81±1.11[a]	2.83±0.75[b]	18.44±0.82[a]

Data are expressed as mol % and are means ± SD of four samples in each group derived from two independent experiments. Statistical analysis was by the one-way ANOVA followed by Tukey's HSD test. Different letters in the same row indicate statistical significance ($p<0.05$).

Table S3. Total FA, PUFA, and DHA content in control (Ctrl) and supplemented cells after 24 h supplementation.

**FA content ($\mu g/10^6$ cells)**	**Ctrl**	**50 μM DHA**	**50 μM DHA +70 μM PRO**	**50 μM DHA +20 μM PCA**
Total FAs other than DHA	51.47 ± 7.28^{a}	42.81 ± 7.25^{a}	41.32 ± 6.56^{a}	46.44 ± 10.36^{a}
Total PUFAs other than DHA	2.07 ± 0.26^{a}	1.43 ± 0.09^{b}	$1.61 \pm 0.16^{a,b}$	2.10 ± 0.41^{a}

Data are means ± SD of four samples in each group derived from two independent experiments and are calculated from previously published values [20]. Statistical analysis was carried out using one-way ANOVA followed by Tukey's HSD test. Different letters in the same row indicate statistical significance ($p<0.05$).

References

20. Ghini, V.; Di Nunzio, M.; Tenori, L.; Valli, V.; Danesi, F.; Capozzi, F.; Luchinat, C.; Bordoni, A., Evidence of a DHA signature in the lipidome and metabolome of human hepatocytes. *Int J Mol Sci* **2017**, *18*, 359.
